# Holden's Anatomy

**Published:** 1887-10

**Authors:** 


HOLDEN'S ANATOMY.—The Dissection of the Human Body, by Luther Holden, late President of the College of Surgeons of England; Consulting Surgeon to St. Bartholomew's and the Foundling Hospitals. Fifth Edition, by John Langton, Surgeon and Lecturer on Anatomy at St. Bartholomew's Hospital and member of the Board of Examiners, Royal College of Surgeons of England. With over two hundred illustrations. Publishers: P. Blakiston, Son & Co., Philadelphia.

This is a concise, clear and practical treatise on Anatomy, and is adapted not only for students, but for members of the medical profession who wish to refresh their anatomical knowledge.

For the dissecting room this work is especially adapted, and must prove of great service in furnishing a work which is better adapted for such study as the cadaver affords than any other in use.

